# Characteristics of atrioventricular block during slow pathway ablation for atrioventricular nodal re‐entrant tachycardia: A comparative study of cryoablation and radiofrequency ablation

**DOI:** 10.1002/joa3.70072

**Published:** 2025-04-30

**Authors:** Shinichi Tachibana, Tetsuya Asakawa, Yuichiro Sagawa, Manabu Kurabayashi, Kazuya Nakagawa, Kaoru Okishige, Shinsuke Miyazaki, Tetsuo Sasano, Yasuteru Yamauchi

**Affiliations:** ^1^ Department of Cardiology Japan Red Cross Yokohama City Bay Hospital Yokohama Kanagawa Japan; ^2^ Department of Cardiology Yamanashi Kosei Hospital Yamanashi Yamanashi Japan; ^3^ Department of Cardiovascular Medicine Institute of Science Tokyo Bunkyoku Tokyo Japan

**Keywords:** atrioventricular block, atrioventricular nodal re‐entrant tachycardia, catheter ablation, cryoablation, slow pathway

## Abstract

**Introduction:**

Slow‐pathway ablation with cryoablation is a useful tool for treating atrioventricular nodal re‐entrant tachycardia (AVNRT). However, reports on the characteristics of atrioventricular block (AVB) during cryoablation are limited. Therefore, we investigated the differences in the appearance of AVB between cryoablation and radiofrequency ablation (RFA).

**Methods:**

This dual‐center retrospective study included 341 patients who underwent slow‐pathway ablation of AVNRT using cryoablation or RFA.

**Results:**

A total of 137 patients underwent cryoablation (CRYO group, *n* = 137), and 204 underwent RFA (RF group, *n* = 204). Transient AVB during slow‐pathway ablation occurred in 33 patients (24.1%) in the CRYO group and 13 patients (6.4%) in the RF group. The time from the beginning of the P‐R interval prolongation to the occurrence of second‐ or third‐degree AVB was significantly longer in the CRYO group (6.6 ± 3.7 s) compared to the RF group (1.2 ± 0.3 s, *p* < 0.01). Three patients in the RF group developed complete AVB requiring pacemaker implantation, whereas none of the patients in the CRYO group developed permanent AVB. After a median follow‐up of 221 ± 186 days, AVNRT recurred in 13 patients (9.5%) in the CRYO group and in 7 patients (3.4%) in the RF group (*p* < 0.01).

**Conclusion:**

Cryoablation gradually induces atrioventricular conduction disturbances when AVB occurs inadvertently, taking longer than RFA. Compared to RFA, cryoablation has a relatively high incidence of transient AVB during slow‐pathway ablation but does not result in permanent AVB.

## Graphical abstract

This study aimed to investigate the differences in the appearance of atrioventricular block (AVB) between cryoablation and radiofrequency (RF) ablation for atrioventricular nodal re‐entrant tachycardia (AVNRT). Our findings revealed that cryoablation for AVNRT has a higher incidence of transient AVB than RF ablation. Cryoablation gradually causes atrioventricular conduction disturbances when AVB occurs inadvertently, which takes longer than RF ablation.

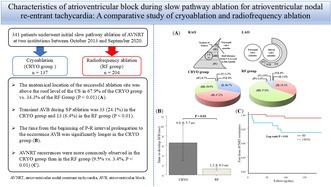

## 1 INTRODUCTION

Radiofrequency ablation (RFA) is highly effective for treating atrioventricular nodal re‐entrant tachycardia (AVNRT),^1^ with an acute success rate of 96.1%–97.3%.^2^, ^3^ However, the incidence of persistent atrioventricular block (AVB) is 0.3%–1.4%.^1^, ^4^ AVB is more likely to occur in the anterior septum during ablation than in the posterior septum because the compact atrioventricular node is closer to the anterior septum. Cryoablation is equally effective and safe as RFA for AVNRT treatment.^5^ Two meta‐analyses have indicated that long‐term recurrence rates are higher with cryoablation than with RFA; however, reports of permanent AVB after cryoablation are limited.^5^, ^6^ A recent multicenter study reported that the incidence of AVB during the cryoablation procedure was 10.1%.^7^ Although it is a well‐established clinical fact that differences exist in the rate of atrioventricular conduction delay during ablation procedures, no previous studies have investigated the detailed progression of AVB development. Therefore, this study aimed to compare the progression time of atrioventricular conduction disturbances during cryoablation and RFA, and the recovery time following AVB occurrence.

## 2 MATERIALS AND METHODS

### 2.1 Study population

This study included 341 consecutive patients aged >15 years who underwent initial catheter ablation for AVNRT at Japan Red Cross Yokohama City Bay Hospital (Yokohama, Japan) and Yamanashi Kosei Hospital (Yamanashi, Japan) between October 2013 and September 2020. All patients experienced palpitations and had paroxysmal supraventricular tachycardia documented on a 12‐lead or Holter electrocardiogram (ECG). Written informed consent for the ablation procedure was obtained from all patients. The study protocol was approved by the hospital's Institutional Human Ethics Committee and complied with the principles of the Declaration of Helsinki.

Antiarrhythmic drugs were discontinued for more than five half‐lives before the ablation procedure. Amiodarone was not administered to any patient prior to the ablation procedure.

### 2.2 Electrophysiology study

All patients underwent a detailed electrophysiological study performed under local anesthesia. Vascular access was obtained through the right internal jugular and femoral veins. Heparin was administered as an intravenous bolus. Multi‐electrode catheters were positioned in the right atrium, coronary sinus (CS), right ventricular apex, and right atrial septum to record the His‐bundle potential. Atrial and ventricular burst pacing was performed to evaluate antegrade or retrograde atrioventricular nodal conduction. Programmed atrial and ventricular stimulation was performed to investigate the dual atrioventricular nodal pathways and the effective refractory period of the fast pathway.

If AVNRT was not inducible, isoproterenol was infused to increase the heart rate, and atrial burst pacing was repeated. The diagnostic criteria and classification of AVNRT were based on standard criteria.^8^ The EnSite system (St. Jude Medical, St. Paul, MN, USA) was utilized for three‐dimensional mapping. The choice of RFA or cryoablation for treating AVNRT depended on the operators' decision.

### 2.3 Radiofrequency ablation procedure

RFA of the slow pathway was performed using a conventional 4‐mm tip catheter, initially applied at the posterior site of Koch's triangle with an atrial‐to‐ventricular potential ratio of 0.1–0.3. When a junctional rhythm did not appear during slow‐pathway ablation at the midseptal or posteroseptal site, the RFA site was gradually shifted from the initial application site or CS toward the anteroseptal site near the His‐bundle potential recording site. Regardless of the AVNRT type, ablation was performed with an optimal atrial‐to‐ventricular potential ratio within Koch's triangle area. Radiofrequency energy was delivered using a generator with a power output of 20–30 W.

The radiofrequency current was stopped when junctional beats were observed, and RFA was restarted at the same site during high‐rate atrial burst pacing, with pacing cycle lengths ranging from 400 to 500 ms to maintain 1:1 fast‐pathway conduction. If faster junctional tachycardia appeared during RFA, radiofrequency energy was immediately discontinued, and no further RFA was applied at that site. Radiofrequency energy was delivered for 60 s at each ablation site. If the P‐R interval was prolonged or AVB occurred during RFA, the radiofrequency current was stopped immediately, and the catheter was simultaneously withdrawn from the ablation site. Programmed atrial stimulation was repeatedly performed within 30 min of the last application to investigate dual pathways with and without isoproterenol administration. RFA was repeated until AVNRT was no longer inducible, and the ablation endpoint was achieved. The endpoint for slow‐pathway ablation, using either RFA or Cryoablation, was defined as the noninducibility of AVNRT or the presence of a single atrial echo beat during atrial double stimulation under isoproterenol administration.

### 2.4 Cryoablation

Cryoablation for AVNRT was performed using a 6‐mm tip cryoablation catheter (Freezor Xtra, Medtronic, Minneapolis, MN, USA). At the start of the ablation procedure, cryomapping (cooling the catheter tip to −30°C) was initiated at a target site in the posterior or middle part of Koch's triangle with an atrial‐to‐ventricular potential ratio of 0.1–0.5. Cryomapping was performed during tachycardia if AVNRT could be easily induced. If AVNRT was difficult to induce or lacked reproducibility, cryomapping was performed during sinus rhythm. During this, the disappearance of slow‐pathway conduction was confirmed using programmed atrial extra‐stimuli. After confirming the elimination of slow‐pathway conduction and ensuring no impairment of fast‐pathway conduction during cryomapping, cryoablation (cooling the catheter tip to −80°C) was performed for 4 min. An additional “insurance” cryoablation was conducted at the same site. During cryoablation, high‐rate atrial burst pacing with 1:1 fast‐pathway conduction was used to promptly detect disturbances in fast‐pathway conduction. The P‐R interval was continuously and meticulously monitored throughout the procedure. If the P‐R interval prolonged by >50 ms or AVB occurred during cryoablation, freezing was immediately stopped, and the catheter was withdrawn. In that case, cryoablation was applied below the site of transient AVB with fluoroscopic and three‐dimensional mapping confirmation. Programmed atrial stimulation was repeatedly performed within 30 min of the last application, both with and without isoproterenol, to confirm the elimination or modification of the slow pathway. Cryoablation was repeated until AVNRT was no longer inducible. The endpoint for the cryoablation procedure was the same as that of the RFA procedure: the noninducibility of AVNRT or the presence of a single atrial echo beat during atrial double stimulation under isoproterenol administration.

### 2.5 Definition

Transient AVB was defined as the development of a temporary second‐ or third‐degree AVB during ablation. Permanent AVB was defined as the development of a second‐ or third‐degree AVB during ablation that required pacemaker implantation. We analyzed the first occurrence of AVB in the eligible patients.

### 2.6 Follow‐up

All patients were discharged without antiarrhythmic drugs. Patients were encouraged to visit the hospital at 1, 3, and 6 months postdischarge. During follow‐up visits, a physical examination, ECG recording, and a review of the patient's symptoms were conducted. Holter ECG monitoring was scheduled within 3 months after the procedure. All patients were instructed to inform their attending physician if they experienced any symptoms suggestive of arrhythmia recurrence. In such cases, rhythm assessment was performed using 12‐lead ECG and Holter ECG examinations. If supraventricular tachycardia was not recorded despite AVNRT‐related symptoms, patients were advised to undergo an electrophysiological study. AVNRT recurrence was defined as its documentation on a 12‐lead ECG, Holter ECG, or electrophysiological study.

### 2.7 Statistical analysis

Continuous variables are expressed as the mean ± SD, and significant differences were analyzed using Student's *t*‐test or the Mann–Whitney *U* test. Categorical data, expressed as numbers and percentages, were compared using the *χ* ^2^ test or Fisher's exact test. Statistical significance was set at *p* < 0.05. AVNRT recurrence was analyzed using the log‐rank test. Statistical analyses were conducted using EZR software (Saitama Medical Center, Jichi Medical University, Saitama, Japan), a graphical user interface for R (The R Foundation for Statistical Computing, Vienna, Austria).

## 3 RESULTS

### 3.1 Baseline characteristics

Table 1 presents the baseline clinical characteristics of the study population. A total of 341 patients (59.5% female; mean age, 58 ± 16 years) underwent initial catheter ablation for AVNRT and achieved the ablation endpoint. Of these, 137 patients underwent cryoablation (CRYO group), and 204 patients underwent RFA (RF group). Typical AVNRT was observed in 288 patients (84.5%) (CRYO 89.1% vs. RF 81.4%, *p* = 0.07). Atypical AVNRT was observed in 53 patients (15.5%), of which fast‐slow and slow‐slow AVNRT was found in 34 (10.0%) and 19 (5.6%) patients, respectively.

**TABLE 1:** Baseline patient characteristics.

Variable	Overall (*n* = 341)	CRYO (*n* = 137)	RF (*n* = 204)	*p* value*
Age, years	57.9 ± 16.5	56.0 ± 17.1	59.1 ± 16.0	0.09
Female, *n*	203 (59.5%)	79 (57.7%)	124 (60.8%)	0.58
Hypertension, *n*	96 (28.2%)	32 (23.4%)	64 (31.4%)	0.11
Diabetes, *n*	28 (8.2%)	8 (5.8%)	20 (9.8%)	0.23
Heart failure, *n*	3 (0.9%)	1 (0.7%)	2 (1.0%)	1.0
Stroke or TIA, *n*	10 (2.9%)	2 (1.5%)	8 (3.9%)	0.33
P‐R interval, ms	161.9 ± 24.1	161.1 ± 23.2	162.4 ± 24.8	0.61
LVEF, %	68.3 ± 6.0	67.9 ± 5.9	68.6 ± 6.2	0.28

*Note*: Values are expressed as the mean ± SD or as *n* (%).

Abbreviations: LVEF, left ventricular ejection fraction; TIA, transient ischemic attack.

* *p* values were compared between the CRYO and RF groups.

No significant differences were observed between the CRYO and RF groups in terms of age, gender, AVNRT type, or comorbidities, including hypertension, diabetes, history of congestive heart failure, or stroke. The P‐R intervals on the 12‐lead ECG and the left ventricular ejection fraction were also comparable between the two groups.

### 3.2 Catheter ablation for slow pathway

Table 2 presents the procedural variables analyzed in this study. The atrio‐Hisian (AH) and Hisian‐ventricle (HV) intervals, as well as the tachycardia cycle length, were similar between the two groups. None of the patients in the CRYO group experienced second‐ or third‐degree transient AVB during cryomapping. However, the incidence of transient AVB was significantly higher in the CRYO group than in the RF group (24.1% vs. 6.4%, *p* < 0.01). No patients in the CRYO group developed permanent AVB, whereas three patients in the RF group required pacemaker implantation because of permanent AVB. At the ablation endpoint, no significant differences were observed between the two groups regarding the complete elimination of slow‐pathway conduction or the presence of a residual slow pathway without a single echo. However, significantly more patients in the RF group exhibited an AH jump with a single echo (*p* < 0.01).

**TABLE 2:** Procedural variables.

Variable	Overall (*n* = 341)	CRYO (*n* = 137)	RF (*n* = 204)	*p* value*
Atypical AVNRT, *n*	53 (15.5%)	15 (10.9%)	38 (18.6%)	0.07
TCL, ms	365 (325, 423)	369 (330, 421)	363 (324, 424)	0.966
*During sinus rhythm*				
AH interval, ms	94.0 ± 24.0	91.5 ± 23.5	96.2 ± 24.4	0.186
HV interval, ms	43.8 ± 7.9	44.0 ± 8.1	43.6 ± 7.8	0.722
Transient AVB, *n*	46 (13.5%)	33 (24.1%)	13 (6.4%)	<0.01
Transient 2nd degree AVB, *n*	24 (7.0%)	15 (10.9%)	9 (4.4%)	0.03
Transient 3rd degree AVB, *n*	22 (6.5%)	18 (13.1%)	4 (2.0%)	<0.01
Permanent AVB, *n*	3 (0.9%)	0 (0%)	3 (1.5%)	0.28
Number of energy applications, *n*	7 (5, 9)	7 (6, 9)	6 (5, 9)	0.1
*Ablation endpoint*				
Elimination of a slow pathway, *n*	238 (69.8%)	102 (74.4%)	136 (66.7%)	0.15
AH jump without an echo, *n*	40 (11.7%)	21 (15.3%)	19 (9.3%)	0.12
AH jump with an echo, *n*	63 (18.5%)	14 (10.2%)	49 (24.0%)	<0.01

*Note*: Values are expressed as the mean ± SD, median (25th, 75th interquartile range), or as *n* (%).

Abbreviations: AH, atrio‐Hisian; AVB, atrioventricular block; AVNRT, atrioventricular nodal reentrant tachycardia; HV, Hisian‐ventricle; TCL, tachycardia cycle length.

* *p* values were compared between the CRYO and RF groups.

**FIGURE 1:**
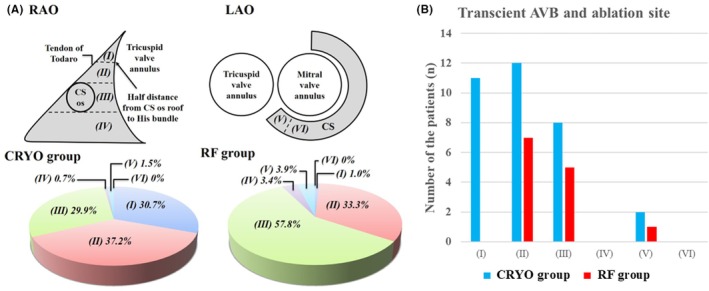
(A) The anatomical location of the successful site was categorized into six regions: (i) The upper part of Koch's triangle, between the coronary sinus (CS) ostial roof and the His‐bundle, (ii) The lower part of Koch's triangle, between the CS ostial roof and the His‐bundle, (iii) The region at the same height as the CS in Koch's triangle, (iv) The region below the bottom of the CS, (v) inside the CS ostium, and (vi) inside the CS. (B) The anatomical location of the ablation site and its relationship to transient AVB.

**FIGURE 2:**
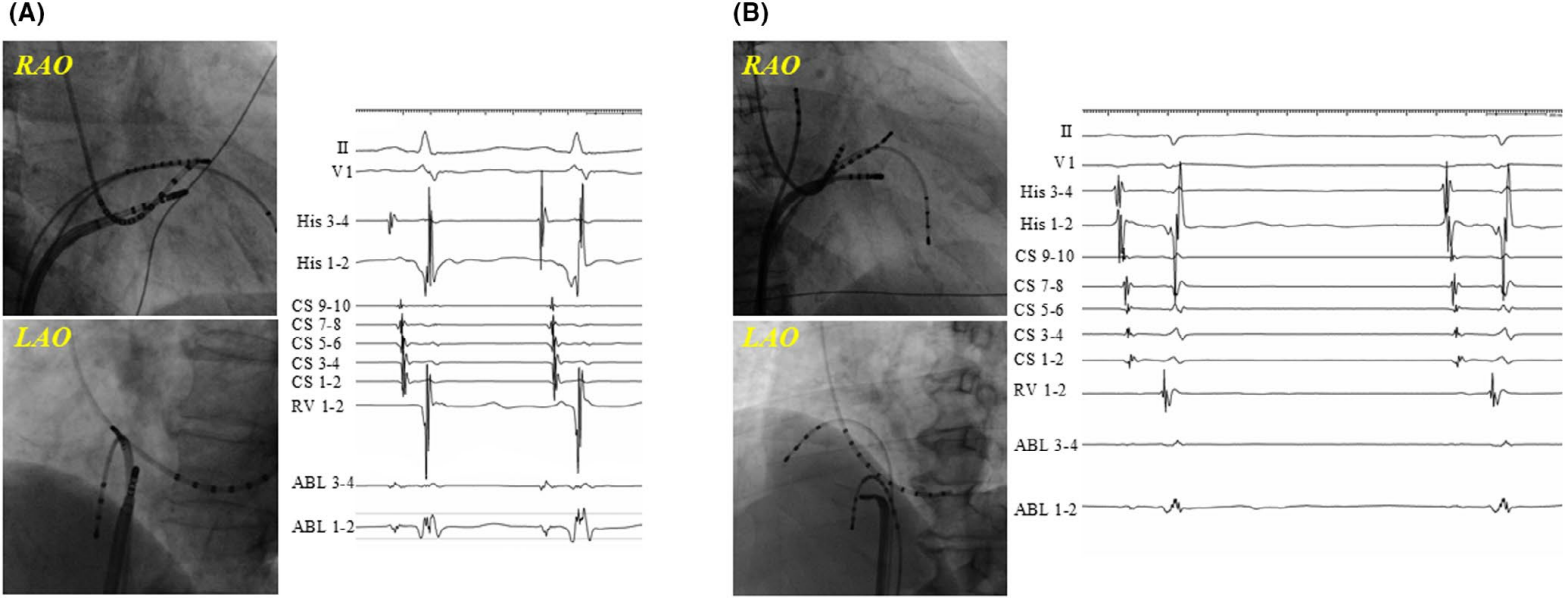
The representative fluoroscopic images and electrograms of the successful ablation sites in the CRYO (A) and RF (B) groups.

Figure 1A shows schematic images of the RAO and LAO view, highlighting the anatomical locations of successful ablation sites. A total of 119 patients underwent a successful ablation in region II (Figure 1): 51 in the CRYO group and 68 in the RF group. Of those, transient AVB occurred in 12 (23.5%) and 7 (10.3%) patients in the CRYO and RF groups, respectively (*p* = 0.09). Moreover, 3 (4.4%) patients in the RF group developed permanent AVB. A log‐rank analysis showed that AVNRT recurrence was comparable between the CRYO and RF groups (*p* = 0.063). In addition, a total of 160 patients underwent a successful ablation in region III (Figure 1): 41 in the CRYO group and 119 in the RF group. Of those, transient AVB occurred in eight (19.5%) and five (4.2%) patients in the CRYO and RF groups, respectively (*p* = 0.006). No patients developed permanent AVB during the slow‐pathway ablation in region III. Among the patients who underwent a successful ablation in region III, a log‐rank analysis showed that there were significantly more AVNRT recurrences in the CRYO group than in the RF group (12.2% vs. 3.4%, *p* < 0.001). Figure 2 shows representative fluoroscopic images and electrograms of the successful ablation sites in the CRYO and RF groups. The successful ablation site was located above the roof level of the CS in 67.9% of patients in the CRYO group compared to 34.3% in the RF group (*p* < 0.01). None of the patients required a septal puncture for slow‐pathway modifications.

### 3.3 AVB during catheter ablation

**FIGURE 3:**
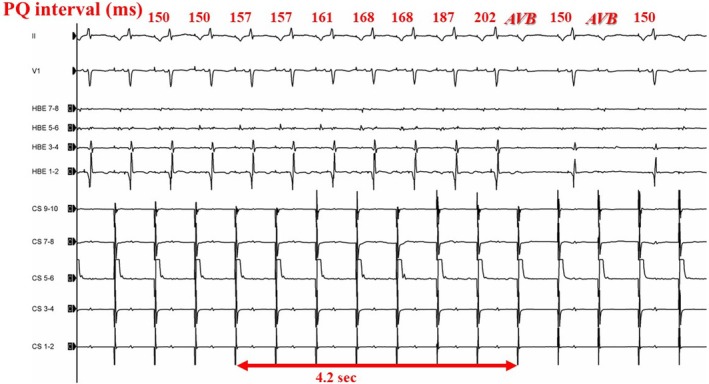
Case of AVB during cryoablation for AVNRT. The P‐R interval gradually prolonged during cryoablation, with AVB occurring at 4.2 s.

**FIGURE 4:**
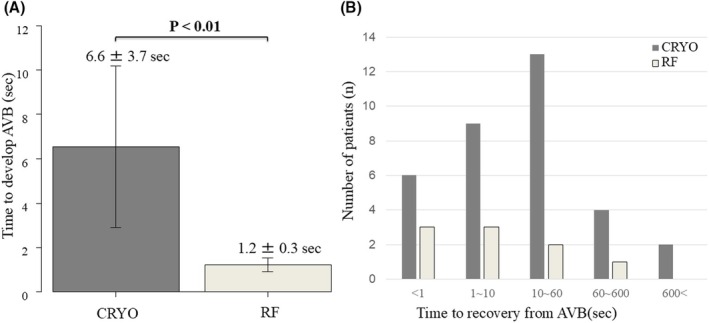
(A) Time to develop AVB. In patients with AVB, the mean time to develop AVB was 6.6 ± 3.7 s in the CRYO group and 1.2 ± 0.3 s in the RF group (*p* < 0.01). (B) time to recover from AVB. In patients with transient AVB, the median time to recovery from AVB was 13 s in the CRYO group and 9 s in the RF group (*p* = 0.70).

Figure 3 illustrates an example of the electrophysiological findings of AVB during cryoablation. The P‐R interval on the 12‐lead ECG progressively increased, eventually leading to AVB. The number of transient AVB cases and the anatomical locations of ablation sites are presented in Figure 1B. The baseline characteristics (such as age, gender, or comorbidities) and AH/HV intervals were comparable between the patients with and without transient AVB. However, the patients with transient AVB had a significantly higher frequency of typical AVNRT than those without (96.7% vs. 82.7%, *p* = 0.042). The atrial‐to‐ventricular potential ratio at the site of transient AVB was 0.23 ± 0.08 in the CRYO group and 0.21 ± 0.05 in the RF group, respectively (*p* = 0.41). Among the patients with AVB, the mean time to develop AVB was 6.6 ± 3.7 s in the CRYO group and 1.2 ± 0.3 s in the RF group (*p* < 0.01) (Figure 4A). Additionally, for patients with transient AVB, the median recovery time was 13 s in the CRYO group and 9 s in the RF group (*p* = 0.70) (Figure 4B). In the CRYO group, the transient AVB lasted up to 1065 s in some cases but resolved intraoperatively in all patients. In contrast, in the RF group, AVB did not resolve intraoperatively in three patients, necessitating the insertion of temporary pacing. These three patients later required permanent pacemaker implantation.

### 3.4 Follow‐up

**FIGURE 5:**
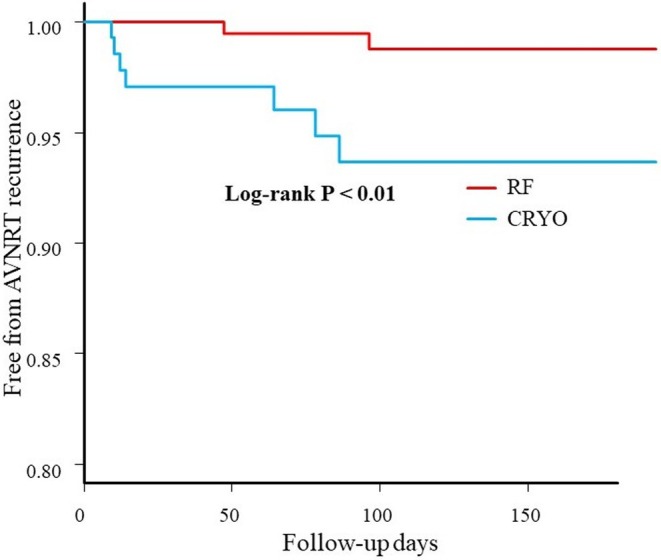
Long‐term freedom from AVNRT recurrence after RFA or cryoablation.

No patients who experienced transient AVB during the procedure developed permanent AVB after discharge. Figure 5 illustrates the Kaplan–Meier curve for freedom from AVNRT recurrence. After a median follow‐up of 221 ± 186 days (range, 28–894 days), AVNRT recurred in 13 patients (9.5%) in the CRYO group and 7 patients (3.4%) in the RF group (*p* < 0.01). Log‐rank analysis revealed no significant difference in AVNRT recurrence based on whether ablation was performed above the CS in either group. There was no significant difference in AVNRT recurrence between the patients with and without transient AVB during the procedure (*p* = 0.145). Additionally, no differences in AVNRT recurrence were observed with different ablation endpoints, including complete elimination of the slow pathway, an AH jump without an echo, or an AH jump with an echo, in both groups. Among the patients with AVNRT recurrence, 12 underwent repeat catheter ablation for slow‐pathway modification.

## 4 DISCUSSION

In this study, 341 patients who underwent RFA or cryoablation for AVNRT were analyzed. The main findings of the study are summarized as follows: (1) in the CRYO group, no patients developed persistent AVB. However, transient AVB, including both second‐degree and third‐degree types, occurred more frequently until reaching the ablation endpoint compared to the RF group. (2) The P‐R interval was prolonged more gradually in the CRYO group, and the time from the onset of prolonged P‐R interval to the development of second‐degree or higher AVB was significantly longer in the CRYO group compared to the RF group. (3) Transient AVB resolved intraoperatively in all patients in the CRYO group, whereas two patients in the RF group developed permanent AVB, requiring pacemaker implantation. (4) AVNRT recurrences were more common in the CRYO group than in the RF group.

### 4.1 AVB characteristics during ablation

In this study, transient AVB occurred in 24.1% of patients who underwent cryoablation, which was significantly higher than the rate observed in patients who underwent RFA. A recent randomized controlled study reported that the incidence of transient first‐ to third‐degree AVB was higher in the CRYO group than in the RF group (25% vs. 5.1%, *p* < 0.01).^9^ Similarly, a North American multicenter prospective study (ICY AVNRT study) reported that 25.2% of patients undergoing cryoablation for AVNRT experienced transient first‐ to third‐degree AVB. Among this group, the highest degree of AVB observed was first‐degree in 31%, second‐degree in 48%, and third‐degree in 21%.^10^ These findings indicate that the incidence of AVB during cryoablation is relatively higher than during RFA. This difference may be attributed to the procedural approach, as cryoablation is typically performed slightly above the ablation site used for radiofrequency ablation because of its perceived safety compared to RFA.

Insulander et al. investigated the acute and long‐term risks of AVB during cryoablation.^11^ They reported that transient first‐ to third‐degree AVB occurred in 12% of patients who underwent cryoablation for substrates adjacent to the atrioventricular node; however, no significant difference was observed in the P‐R interval before and after the procedure.^11^ The study concluded that transient AVB during cryoablation is a benign finding.^11^

In our study, the time from P‐R interval prolongation to the onset of AVB was significantly shorter in the RF group, averaging 1.2 ± 0.3 s, compared to 6.6 ± 3.7 s in the CRYO group. To our knowledge, no previous study has investigated the time from P‐R prolongation to the onset of second‐ or third‐degree AVB. AVB during slow‐pathway ablation reflects unfavorable or unexpected fast‐pathway disturbances. In the present study, the difference in atrioventricular conduction disturbances between the CRYO and RF groups was demonstrated by comparing the time to the occurrence of AVB. Cryoablation results in a more gradual progression of AVB than RFA. Even if the P‐R interval prolongs, increasing the risk of AVB, its slower progression allows for safer ablation closer to the His‐bundle. Moreover, it has been demonstrated that even if AVB occurs, it can always be reversed by immediately stopping the cooling process. For reference, a prior experimental study examined the histological changes associated with AVB occurrence as a function of cryoablation duration.^12^ The study found that atrioventricular nodal conduction fully recovered in animals when freezing was stopped within 10 s of complete AVB onset during cryoablation.^12^ Moreover, the likelihood of persistent AVB increased with the duration of cryoablation after AVB onset. Persistent AVB was observed in 25%, 75%, and 100% of animals with cryoablation durations of 20, 40, and 60 s following AVB onset, respectively.^12^ These findings suggest that AVB is not irreversible if cryoablation is interrupted promptly after its occurrence. As AVB is reversible even when it develops, cryoablation should be considered in patients requiring ablation near the His‐bundle electrogram. In this study, the safety of cryoablation for AVB was assessed by observing its progression. The gradual onset of AVB was demonstrated, further supporting the safety of cryoablation. Consequently, cryoablation appears to be significantly safer than RFA for slow‐pathway modification, particularly in patients with small Koch's triangles, which are more common in older adults.

### 4.2 Successful ablation site

More patients in the CRYO group achieved successful ablation above the level of the CS compared to those in the RF group, contributing to a higher incidence of transient AVB. Wells et al. reported that the anatomical location of a successful ablation site during cryoablation was above the CS level in 60% of participants.^10^ Operators often initiate ice mapping and cryoablation near the His‐bundle potential recording site, as there have been no reported cases of permanent AVB following cryoablation for AVNRT.^5^, ^6^

In our study, cryoablation was initially performed at a target site in the posterior or middle part of Koch's triangle, with an atrial‐to‐ventricular potential ratio of 0.1–0.5. However, when cryoablation in the posterior or middle part of Koch's triangle failed to eliminate or modify the slow pathway, the ablation site was gradually shifted toward the mid‐septal and anterior‐septal regions. The recent study demonstrated that the collision site of delayed atrial activation in Koch's triangle could be a successful ablation site based on ultra‐high‐density mapping. Although the ultra‐high‐density mapping was not available in this study, it may have allowed us to reduce the number of applications, resulting in decreased transient AVB.^13^

In the CRYO group, 30.7% were successfully ablated in the high Koch triangle area (Figure 1, region I) compared with 1.0% in the RF group (Figure 1A). Therefore, the incidence of AVB during slow‐pathway ablation was significantly higher in the CRYO group than in the RF group. Cryoenergy can be safely delivered at sites with higher atrioventricular potential ratios than those typically associated with successful RFA.^10^, ^14^ The present study examined the ablation site at the time of block appearance and compared it between cryoablation and RFA. No previous studies have compared AVBs that appear during cryoablation or RFA for AVNRT. Our findings also demonstrate that cryoablation can be safely performed in anatomical regions where RFA is associated with a high risk of permanent AVB.

## 5 STUDY LIMITATIONS

This study had several limitations. First, it was a retrospective analysis. A prospective multicenter study is necessary to validate our findings. In particular, the number of eligible patients in the CRYO group was small, and a larger sample size is required to prove the safety of cryoablation for AVNRT. Second, the follow‐up period was relatively short. A recent study reported that AVNRT recurrence occurred much later after cryoablation than after RFA,^15^ and many patients who underwent cryoablation developed AVNRT recurrence beyond the follow‐up period of our study. Third, the target atrial‐to‐ventricular potential ratio was the same for both the CRYO and RF groups. Wells et al. reported that cryoablation could be successfully performed at sites with a higher atrial‐to‐ventricular potential ratio compared to RFA.^10^ In our study, cryoablation was applied more extensively than RFA, which may have influenced the outcomes. Fourth, a previous study identified prolonged time to reach −70°C and higher minimum temperatures at the cryoablation catheter tip as independent risk factors for AVNRT recurrence.^16^ In our study, cooling dynamics were not investigated; however, they may have contributed to the transient AVB observed during cryoablation. Fifth, a conventional 4‐mm tip catheter was utilized in the present study. An irrigated RF ablation catheter was shown to be feasible and safe and could avoid ablation in close proximity to the His‐bundle region.^17^ Not using an irrigation catheter might be related to the occurrence of AVB and clinical outcome in the RF group.

## 6 CONCLUSIONS

Cryoablation for AVNRT has a higher incidence of transient AVB compared to RFA. However, atrioventricular conduction disturbances during cryoablation progress gradually, with a longer time from P‐R prolongation to second‐ or third‐degree AVB than RFA. This suggests that cryoablation may offer a safety advantage, especially for patients at a higher risk of permanent conduction disturbances.

## FUNDING INFORMATION

This research did not receive any specific grants from funding agencies in the public, commercial, or not‐for‐profit sectors.

## CONFLICT OF INTEREST STATEMENT

Authors declare no conflict of interests for this article.

## ETHICS STATEMENT

Approval of the Research Protocol: The hospital's institutional human ethics committee approved the study protocol.

Informed Consent: Written informed consent was provided to all patients.

Registry and the Registration No. of the study/trial: 2020–36.

Animal Studies: N/A.

## Data Availability

The data underlying this article will be shared on reasonable request to the corresponding author.
